# Prognostic analysis of telangiectatic osteosarcoma of the extremities

**DOI:** 10.3389/fonc.2022.1105054

**Published:** 2023-02-06

**Authors:** Wei Zhong, Wei Luo, Zili Lin, Ziyi Wu, Yuhao Yuan, Yizhe He

**Affiliations:** ^1^ Department of Orthopaedics, Xiangya Hospital, Central South University, Changsha, China; ^2^ National Clinical Research Center for Geriatric Disorder, Xiangya Hospital, Changsha, China

**Keywords:** telangiectatic osteosarcoma, SEER, survival prognosis, limb salvage, local inflammation, neoadjuvant chemotherapy

## Abstract

**Background and objectives:**

Telangiectatic osteosarcoma (TOS) is a rare but highly malignant subtype of osteosarcoma. Although surgical treatment is the primary treatment modality for osteosarcoma, evidence on the benefits of different surgical methods in patients with TOS is lacking. This study aimed to compare the effects of different surgical and adjuvant treatments on overall survival of TOS, and the association of patient demographics, oncological characteristics, and socioeconomic status on treatment outcomes.

**Method:**

This retrospective study selected the most common TOS cases of the extremities registered in the Surveillance, Epidemiology, and End Results (SEER) database from 1989 to 2019. Univariate and multivariate Cox regression models were used to analyze all prognostic factors, and Kaplan-Meier analyses were performed for disease-specific treatment factors of survival.

**Result:**

A total of 127 patients were included in the analysis. The average age at initial diagnosis was 20.09 years. In univariate analyses, the absence of metastasis at initial diagnosis, limb-salvage surgery, adjuvant chemotherapy, and no regional lymph node dissection were associated with a lower risk of death. Multivariate analysis further showed that the presence or absence of distant metastasis and regional lymph node dissection, implementation of adjuvant chemotherapy, and choice of surgical method were independent predictors of prognosis.

**Conclusion:**

Distant metastasis and regional lymph node dissection are associated with poorer outcomes in TOS, and amputation has no better prognosis than limb salvage surgery. Compared with conventional chemotherapy, neoadjuvant chemotherapy did not significantly improve the prognosis of TOS.

## Introduction

Osteosarcoma is a malignancy originating from mesenchymal tissue (1) that has an extremely poor prognosis owing to its high degree of malignancy and high invasiveness (2). Osteosarcoma is classified into eight subtypes according to its histological type and imaging features as follows: conventional, telangiectatic, small-cell, low-grade central, secondary, parosteal, periosteal, and high grade. In addition to conventional osteosarcoma, other types of osteosarcomas are also known as non-conventional osteosarcoma (3, 4). Telangiectatic osteosarcoma (TOS) is a nonconventional osteosarcoma that was first discovered by Paget in 1853 (5). It is a relatively rare type of osteosarcoma, accounting for less than 4% of all cases (6). TOS is primarily diagnosed by imaging examination and biopsy. At the early stage, single or multiple hemorrhagic cysts containing blood, bony septa, and a small amount of solid tumor tissue or necrotic tissue can be found in the tumor (7). The distribution of the primary site of TOS is similar to that of conventional osteosarcoma. It tends to occur in long tubular bones, with the femur being the most involved, followed by the tibia and humerus. In these bones, the metaphysis is the most common site of origin. These cases that originate in the extremities account for more than 90% of all TOS, and those originating in other bones such as the pelvis, sternum, ribs, and mandible have rarely been reported (8).

TOS originally has poor prognosis, but some studies have reported that the long-term overall survival rate of TOS has improved from less than 20% to approximately 60% since the introduction of neoadjuvant chemotherapy (9). However, owing to the lack of controlled and large-scale studies, the efficacy of neoadjuvant chemotherapy still needs to be further evaluated. In addition, surgical treatment, the main method of osteosarcoma treatment, remains a mainstay. Currently, evidence on the benefits of different surgical methods in patients with TOS is lacking.

Thus, this study aimed to identify specific prognostic factors for survival, both positive and negative, in TOS to improve and guide diagnosis and treatment in the future. Towards this goal, the epidemiology and survival data of patients with TOS in the extremities and who received surgical treatment were collected from the Surveillance, Epidemiology, and End Results (SEER) database.

## Materials and methods

The SEER database is the most important source of information on cancer incidence and survival rates in the United States. It has registries in 18 states and counties in the United States, covering 35% of the US population. It not only covers a wide population and area, but also has high data integrity for recording various clinical information of tumor patients. SEER requires cancer registries to achieve at least a 95% follow-up rate. A review of the most recent database reports from 1975 to 2016 found that follow-up rates were over 98% and 97% for male and female patients, respectively. Given these advantages, the SEER database is convenient for the study of rare tumors (10).

**Figure 1:**
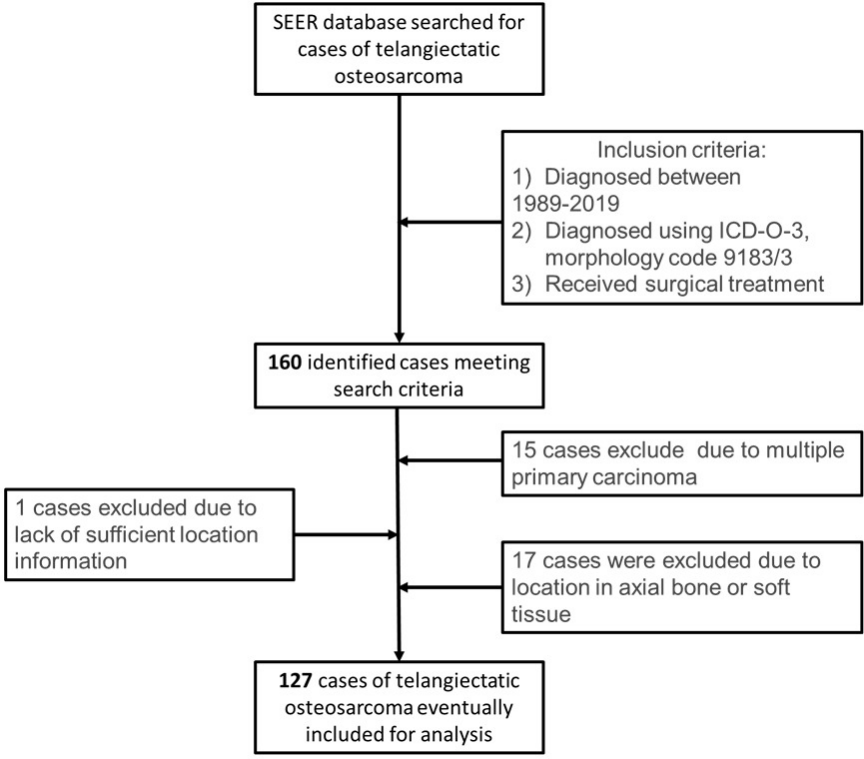
Patient selection flow chart.

Patient information was extracted from the SEER database using the International Classification of Cancer Diseases, Third Edition morphological code 9183/3. The inclusion criteria were as follows: (1) diagnosis between 1989 and 2019, (2) a final pathological diagnosis of TOS, and (3) surgical treatment. Among the 160 eligible patients, 33 patients were excluded owing to soft tissue osteosarcoma (n=6), trunk or skull osteosarcoma (n=11), unknown tumor location (n=1), and multiple carcinomas *in situ* (n=15). Finally, 127 patients were included in the analysis. Information on demographic characteristics, oncological characteristics (tumor location, size, grade, and stage at initial diagnosis), treatment (surgery and adjuvant therapy), socioeconomic status (median household income, residence, and the era of treatment), survival time (in months), and ending events was collected from the database (**Figure 1**).

Patient age was converted to categorical variables (0–20 years, 21–40 years, ≥41 years) according to the age distribution trend of the population. Tumor size was converted to a categorical variable (<8 cm or ≥8 cm). The primary tumor location was divided into two groups: the upper and lower extremities. Tumor grade was divided into grades I/II and III/IV according to the degree of tumor differentiation, and tumor stage was classified as localized, regional, and distant according to whether it traversed the compartment or developed distant metastasis. The surgical approach was determined as amputation, limb salvage, and unknown. Adjuvant therapies such as chemotherapy were also administered. The time between initial diagnosis and recent follow-up or all-cause death was recorded to calculate the survival rate in the population, and Kaplan-Meier survival curves were plotted to identify disease-specific prognostic factors. Pearson’s chi-square test and Cox regression analysis were used to determine the association between these variables and overall survival rate, with P<0.05 considered to be statistically significant. After confirming that the equal proportional hazards model was met, Cox multivariate analysis was performed based on the results of the univariate analysis to test prognostic factors associated with overall and disease-specific mortality. All statistical analyses and data visualization were performed using R software version 4.2.0.

## Results

In total, 63 (49.6%) and 74 (51.4%) patients were female and male, respectively. The mean patient age was 20.09 ± 15.09 years. The majority of patients were non-Latino white (40.9%), followed by Latinos (34.6%). Lower extremity tumors (78.0%) were significantly more frequent than were upper extremity tumors (22.0%). In most cases, the tumors were limited to a regional area (beyond compartment) (44.1%), while localized tumors (within compartment) (39.4%) and metastatic tumors (12.6%) were relatively rare. Overall, 41 patients (32.3%) underwent amputation, 84 patients (66.1%) underwent limb salvage, and 2 patients (1.6%) had unknown surgical procedures. Further, 14 (11%) patients underwent additional regional lymph node dissection. Most patients (n=121, 95.2%) received adjuvant chemotherapy; among them, 19 (15.7%) received neoadjuvant chemotherapy alone, 47 (38.8%) received full-course chemotherapy, 7 (5.8%) patients were treated with conventional chemotherapy postoperatively, and 51 patients (42.1%) had unknown sequence of chemotherapy. With respect to the distribution of diagnosis by year, there were 50 cases (39.4%) from 2010 to 2019, 59 cases (46.5%) from 2000 to 2009, and 18 cases (14.2%) from 1989 to 1999. The socio-economic survey of the background of the study population showed that majority of the patients were urban dwellers (88.2%). In addition, a large proportion of the patients had high income, with 63.0% and 29.1% of them having median household incomes of more than $45,000 and $75,000, respectively, and only 7.1% had low incomes (**Table 1**).

**Table 1:** Patient characteristics by surgical approach.

Surgical methods	AmputationN=41	Limb salvage N =84	Unknown N =2
Sex			
Male	29 (70.7%)	43 (51.2%)	2 (100%)
Female	12 (29.3%)	41 (48.8%)	0 (0%)
Race			
Hispanic	15 (36.6%)	29 (34.5%)	0 (0%)
Non-Hispanic White	16 (39.0%)	34 (40.5%)	2 (100%)
Black	7 (17.1%)	12 (14.3%)	0 (0%)
Asia or Pacific Islander	3 (7.3%)	9 (10.7%)	0 (0%)
Age			
0–20	29 (70.7%)	64 (76.2%)	2 (100%)
21–40	7 (17.1%)	16 (19.0%)	0 (0%)
≥41	5 (12.2)	4 (4.8%)	0 (0%)
Primary site			
Upper limb	9 (22.0%)	17 (20.2%)	2 (100%)
Lower limb	32 (78.0%)	67 (79.8%)	0 (0%)
Pathological grade			
I/II	2 (4.9%)	1 (1.2%)	0 (0%)
III/IV	30 (73.2%)	63 (76.8%)	0 (0%)
Unknown	9 (22.0%)	18 (22%)	2 (100%)
Tumor size			
Localized	13 (31.7%)	35 (41.7%)	2 (100%)
Regional	22 (53.7%)	34 (40.5%)	0 (0%)
Distant	6 (14.6%)	10 (11.9%)	0 (0%)
Unknown	0 (0%)	5 (6.0%)	0 (0%)
Regional LN dissection			
Yes	5 (12.2%)	9 (10.7%)	0
No	35 (85.3%)	74 (88.1%)	1 (50%)
Unknown	1 (2.4%)	1 (1.2%)	1 (50%)
Chemotherapy			
Yes	39 (95.1%)	80 (95.2%)	2 (100%)
No	2 (4.9%)	4 (4.8%)	0 (0%)
Unknown	0 (0%)	0 (0%)	0 (0%)
Chemotherapy sequence			
Preoperative	6 (14.6%)	12 (14.3%)	1 (50.0%)
Postoperative	2 (4.9%)	5 (6%)	0 (0%)
Full course	13 (31.7%)	34 (40.5%)	0 (0%)
None	1 (2.4%)	2 (2.4%)	0 (0%)
Unknown	19 (46.3%)	31 (36.9%)	1 (50.0%)
Year of diagnosis			
1989–1999	10 (19.5%)	9 (11.9%)	0 (0%)
2000–2009	22 (53.7%)	35 (41.7%)	2 (100%)
2010–2019	11 (26.8%)	39 (46.4%)	0 (0%)
Residence			
Metro	36 (88.8%)	74 (88.1%)	2 (100%)
Rural	5 (12.2%)	8 (9.5%)	0 (0%)
Unknown	0 (0%)	2 (2.4%)	0 (0%)
Median household Income			
$0–$45,000	2 (4.9%)	7 (8.3%)	0 (0%)
$45,000–$74,999	27 (65.9%)	52 (61.9%)	1 (50.0%)
≥$75,000	12 (29.3%)	24 (28.6%)	1 (50.0%)
Unknown	0 (0%)	1 (1.2%)	0 (0%)

Univariate analysis of overall survival according to population characteristics showed that the survival rate was significantly higher in patients who received limb salvage therapy than in patients who underwent amputation (HR: 0.51, 95% CI: 0.26–1.00, P<0.05) and in patients who received chemotherapy than in those who did not (HR: 0.22, 95% CI: 0.07–0.74, P=0.01). However, there was no evidence of an association with the sequence of adjuvant chemotherapy (postoperative vs. preoperative: HR: 4.7, 95% CI: 0.78–28.18, P=0.09; full course vs. preoperative: HR: 1.87, 95% CI: 0.40–8.67, P=0.42). The prognosis of patients with additional regional lymph node dissection was significantly worse than that of other patients (HR: 4.47; 95% CI, 2.08–9.63; P<0.001). Distant metastasis was also a poor prognostic factor (HR: 2.65; 95% CI, 1.23–5.73; P=0.01). Meanwhile, there were no significant differences in survival according to sex, race, age, era of treatment, tumor size (≥8 cm), and socioeconomic status (income, residence, and treatment era) (**Table 2**).

**Table 2:** Univariate analysis of the effect of patient characteristics on prognosis.

	HR (95% CI)	P
Sex		
Female (vs. Male)	0.87 (0.44–1.73)	0.69
Race		
Hispanic (vs. Non-Hispanic White)	0.84 (0.38–1.87)	0.67
Asia/Pacific Islander (vs. Non-Hispanic White)	1.19 (0.39–3.58)	0.76
Black (vs. Non-Hispanic White)	1.20 (0.46–3.09)	0.71
Age		
21–40 years (vs. 0–20 years)	1.10 (0.45–2.74)	0.83
41+ years (vs. 0~20 years)	0.58 (0.20–1.66)	0.31
Primary site		
Upper limb (vs. Lower limb)	1.07 (0.49–2.36)	0.86
Tumor size		
≥ 8 cm (vs. <8 cm)	1.06 (0.51–2.20)	0.87
Tumor stage		
Localized (vs. Regional)	0.47 (0.19–1.14)	0.09
Distant (vs. Regional)	2.65 (1.23–5.73)	**0.01**
Surgical type		
Limb salvage surgery (vs. Amputation)	0.51 (0.26–1.00)	**<0.05**
Regional lymph node dissection		
Yes (vs. No)	4.47 (2.08–9.63)	**<0.001**
Chemotherapy		
Yes (vs. No)	0.22 (0.07–0.74)	**0.01**
Systemic therapy sequence		
Postoperative (vs. Preoperative)	4.7 (0.78–28.18)	0.09
Full course (vs. Preoperative)	1.87 (0.40–8.67)	0.42
Year of diagnosis		
2000–2009 (vs. 1989–1999)	1.10 (0.45–2.74)	0.83
2010–2019) vs. 1989–1999)	0.58 (0.20–1.66)	0.31
Residence		
Rural (vs. Urban)	1.24 (0.44–3.51)	0.69
Median household Income		
$45,000–$74,999 (vs. $0–$45,000	0.87 (0.26–2.89)	0.82
$75,000+ (vs. $45,000 –$74,999)	0.53 (0.14–2.04)	0.36

P values are calculated based on the Cox hazard proportional hazards model, and P values in bold indicate P<0.05.

HR, disease hazard ratio.

**Figure 2:**
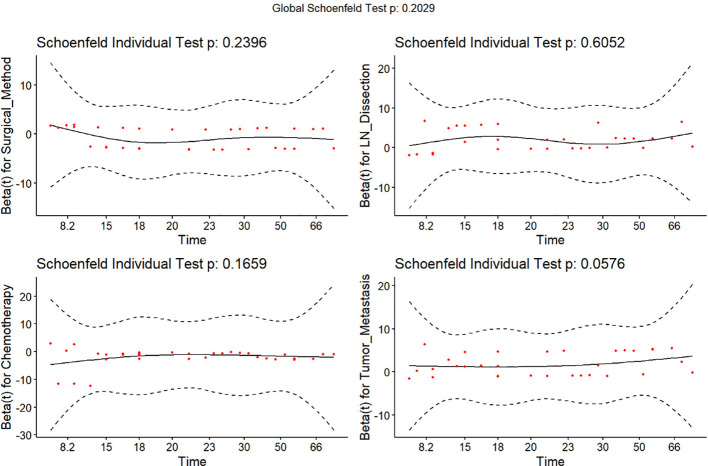
Schoenfeld residual plots to test whether relevant factors match an equal-proportional-hazards model: There was no significant correlation between the candidate variables and time (P>0.05), so the risk ratio between all these variables and outcome was fixed.

The above factors, which had a significant effect on the survival and prognosis of patients, were tested using the equal-proportional hazard hypothesis (**Figure 2**), and the results showed that all these factors match the equal-proportional hazard hypothesis.

**Figure 3:**
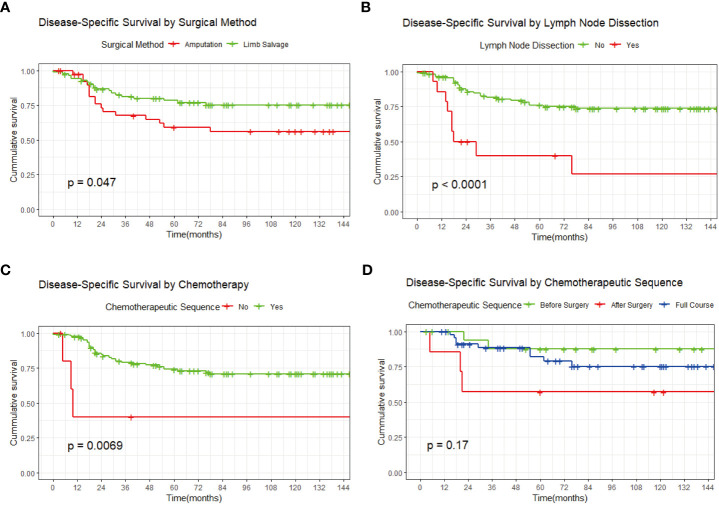
Kaplan-Meier survival curves. **(A)** By surgical method. **(B)** By additional regional lymph node dissection. **(C)** By adjuvant chemotherapy. **(D)** By chemotherapy sequence.

Kaplan-Meier survival analysis according to the treatment factors showed that the overall survival rate was significantly better (1) in patients who received limb salvage therapy than in patients who received amputation and (2) in those who received chemotherapy than in those who did not (**Figures 3A, C**). In contrast, the prognosis of patients with additional regional lymph node dissection was significantly worse than that of patients without this dissection (**Figure 3B**). Meanwhile, the chemotherapy sequence (preoperative, postoperative, or full course) had no significant effect on survival (**Figure 3D**).

Multivariate analysis of disease-specific survival according to surgical method, adjuvant chemotherapy, regional lymph node dissection, and distant metastases is shown in **Table 3**. After controlling for other factors, surgical methods, adjuvant chemotherapy, regional lymph node dissection, and distant metastases were identified to be independent influencing factors of survival prognosis.

**Table 3:** Multivariate analysis of the effects of population characteristics on prognosis.

	HR (95% CI)	P
Tumor stage		
Metastasis (vs. No metastasis)	5.64 (2.53–12.58)	**<0.001**
Surgical type		
Limb salvage surgery (vs. Amputation)	0.47 (0.24–0.94)	**0.03**
Regional lymph node dissection		
Yes (vs. No)	6.92 (2.95–16.22)	**<0.001**
Chemotherapy		
Yes (vs. No)	0.14 (0.04–049)	**0.002**

P values are calculated based on the Cox hazard proportional hazards model, and P values in bold indicate P<0.05.

HR, hazard ratio.

## Discussion

TOS is a high-grade osteosarcoma that accounts for only a small proportion of all osteosarcoma cases; therefore, animal studies and case reports are the primary sources of information regarding this disease (11). The prognosis of TOS is poor, but it has recently improved with the introduction of adjuvant chemotherapy. Some studies reported that TOS has a good response to surgery with adjuvant chemotherapy, but there are few reports on whether there is a difference in the efficacy between preoperative and postoperative chemotherapy (12, 13). Limb-salvage surgery is widely applied for the surgical treatment of osteosarcoma (14, 15). However, as a rare subtype of osteosarcoma, TOS has a high degree of malignancy, unclear borders, and often invades the adjacent soft tissues (2, 12). Complete resection is more difficult than those for other types of osteosarcoma, and there is no evidence that limb salvage can improve patient survival. Given that this tumor is rare, it is difficult to collect information from patients who receive short-term treatment, and prospective studies are not feasible. Thus, we performed a comprehensive analysis of the TOS cases in the SEER database to better understand the characteristics of this osteosarcoma subtype.

The results showed that most of the TOS cases were distributed in the extremities, accounting for 90% of all cases, and the patients were significantly younger (20.09 years vs 40.44 years) than those with TOS in the trunk as the primary site. This is consistent with previous reports (8, 16). Considering the high probability of radical cure by surgery and the patients’ strong will to live, the study selected TOS patients with the limbs as the primary site and who received surgical treatment. In addition to general characteristics, such as race, sex, age, and primary site as study variables, the analysis also evaluated the effects of surgical methods, adjuvant chemotherapy, and other treatment factors on prognosis. With respect to the effect of the surgical procedures, unexpectedly, 14 (11%) patients had undergone additional regional lymph node dissection. The traditional view is that osteosarcoma mainly metastasizes through the blood vessels and rarely through the lymph nodes (17). However, it is unclear whether this finding indicates that lymphatic metastasis is more frequent in TOS than in conventional osteosarcoma. Therefore, the indication for regional lymph node dissection was included in the analysis. Given the long treatment cycle and high cost of osteosarcoma, the rate of treatment compliance is low. The patients’ socioeconomic status is an important factor that may affect prognosis. Therefore, we also examined socioeconomic factors such as treatment era, median household income, and area of residence. Univariate analysis was used to screen the factors affecting survival prognosis, and multivariate analysis was used to further identify the independent prognostic factors under the condition of an equal proportional hazards model.

A total of 127 TOS patients were included in the analysis. Among them, 41 patients (32.3%) underwent amputation, 84 patients (66.1%) underwent limb salvage, and 2 patients (1.6%) had unknown surgical procedures. Most of the patients (n=121, 95.2%) received adjuvant chemotherapy, among them, 19 (15.7%) received neoadjuvant chemotherapy alone, 47 (38.8%) received full-course chemotherapy, 7 (5.8%) patients were treated with conventional chemotherapy postoperatively. The chemotherapy sequence for the rest 51 patients (42.1%) was unknown. Further, race, sex, age, primary site, and all socioeconomic factors had no significant effect on survival prognosis. The overall survival rate was better in patients who received chemotherapy than in those who did not (HR: 0.22, 95% CI: 0.07–0.74, P=0.01). However, the chemotherapy sequence (preoperative, postoperative, or full course) had no significant effect on survival. Interestingly, survival was significantly higher in patients who received limb salvage therapy than in patients who underwent amputation (HR: 0.51, 95% CI: 0.26–1.00, P<0.05). In contrast, the prognosis of patients with additional regional lymph node dissection was significantly worse than that of patients without this dissection. There was no significant difference in the overall survival rate of tumors within the compartment and beyond the compartment. But compared with the former two, distant metastasis was associated with significantly worse prognosis.

Conventional osteosarcoma is highly sensitive to chemotherapy, and thus, chemotherapy is an effective modality for this malignancy. Imaging findings such as tumor shrinkage, clear boundaries, and necrosis can often be observed during neoadjuvant chemotherapy. Patients with osteosarcoma treated with adjuvant chemotherapy usually have a better prognosis than those without. Patients with TOS rarely have similar imaging findings and sometimes show poor imaging findings during chemotherapy (18). Such imaging findings can be misleading and be interpreted as chemotherapy being ineffective for TOS. However, the current study shows that chemotherapy is meaningful for TOS, and patients who received chemotherapy had better overall survival than did those who did not. These findings are consistent with those of previous studies wherein the 5-year survival rate of TOS patients treated with adjuvant chemotherapy was close to that of patients with conventional osteosarcoma (19, 20). Angelini et al. also found that the chemotherapy response rate was an important independent risk factor for overall survival in TOS patients (OR=0.24, 95% CI: 0.11–0.54, P<0.001); patients with poor response to chemotherapy often have poor survival prognosis (9, 21). Meanwhile, the current study found that the chemotherapy sequence (preoperative, postoperative, or full course) had no significant effect on survival, and there was no significant difference in survival prognosis between patients treated with immediate surgery and patients who underwent adjuvant chemotherapy preoperatively. Compared with conventional chemotherapy, neoadjuvant chemotherapy did not significantly improve the prognosis. These need to be further clarified by a larger sample of research. However, the development of genome sequencing and targeted drugs bring sarcoma therapy to precision times. Some new therapies have been used to treat osteosarcoma, such as anti-angiogenesis therapy, molecular targeted therapies, immune-based therapies, etc., which have improved the prognosis of patients with osteosarcoma (22). Combined with these methods, the efficacy of neoadjuvant chemotherapy for TOS is expected to be improved. At present, in view of the progress of many TOS during neoadjuvant chemotherapy, surgery should be performed if the tumor can be completely removed with a negative margin during one-stage surgery.

In total, 84 patients in the current study underwent limb salvage, while 41 patients underwent amputation. The rate of limb salvage surgery in TOS is only 67.2%, and, it is only 78.0% even in the last 10 years. During all periods, the rate of limb salvage for TOS is significantly lower than that for overall osteosarcoma (23–25). TOS amputation is usually performed for because the bone in the TOS lesion is destroyed and completely dissolved, and its boundary is unclear without surrounding bone sclerosis. A considerable proportion of cases are complicated by pathological fractures (9, 13). Neoadjuvant chemotherapy cannot ameliorate these pathological changes. However, TOS is often misdiagnosed as an aneurysmal bone cyst and giant cell tumor of the bone (18, 26, 27). Often, the tumor has already progressed upon correct diagnosis (28). The above two reasons may explain for the higher rate of amputation in TOS than in conventional osteosarcoma. For patients with classic osteosarcoma, the survival rates are the same between limb salvage and amputation (29–31). However, the current study showed that the survival rate was significantly higher in patients who received limb salvage therapy than in those who underwent amputation. One possible explanation is that amputation is often used for patients with vascular and nerve involvement, while limb salvage is usually used for patients without neurovascular involvement. This indicated that the amputation patients may have a more severe condition than limb salvage patients and that there is a certain selection deviation. Therefore, the survival rate of limb salvage is not inferior to that of amputation, and amputation is not the first-line surgical method.

Lymph node metastasis is rare in patients with classical osteosarcoma; therefore, lymph node dissection is not required. In the current study, 11% (14/127) of patients underwent lymph node dissection, and the prognosis of patients who underwent additional regional lymph node dissection was significantly worse than those without addition dissection. Interestingly, none of these cases showed lymphatic metastasis on postoperative pathological examination. It is suggested that lymph node enlargement is more likely to be related to local inflammatory reactions, and this type of local inflammatory reaction might have negative effects on survival prognosis, suggesting that the status of regional lymph nodes has the potential to be an indicator of poor prognosis. This indicates that for TOS patients, lymph node metastases are rare, and lymph node dissection is unnecessary (32). Therefore, lymph node dissection is not recommended in patients with TOS.

This study has some limitations. First, the study was susceptible to selection bias owing to its retrospective nature. Furthermore, as the SEER database did not include chemotherapy response rates and specific chemotherapy regimens, the effects of these factors on patient outcomes were not analyzed. Finally, the SEER database is based on the clinical follow-up data of cancer patients collected from multiple registries across the United States. There is currently a debate on whether the data collected from different registries are significantly heterogeneous, and whether the conclusions apply to populations in other countries or regions. Despite these limitations, this study provides new insights that will help to guide follow-up studies on TOS as a rare disease.

In conclusion, for patients with TOS, the absence of metastasis at initial diagnosis, limb-salvage surgery, adjuvant chemotherapy, and no regional lymph node dissection were associated with a lower risk of death. Distant metastasis and regional lymph node dissection are associated with poorer outcomes. Chemotherapy can significantly improve survival, and neoadjuvant chemotherapy did not significantly improve the prognosis compared with conventional chemotherapy. Besides, amputation has no better prognosis than limb salvage surgery for patients with TOS. Thus, limb salvage surgery is recommended as the first choice of treatment for locally resectable TOS. Finally, regional enlarged lymph nodes are not related to lymphatic metastasis, and lymph node dissection is not recommended for patients with TOS.

## Data availability statement

The original contributions presented in the study are included in the article/**Supplementary Material**. Further inquiries can be directed to the corresponding author.

## Author contributions

WZ designed this study; ZL and ZW extracted data from SEER database; WZ, YY and YH completed the data analysis of this study; WL helped in revision and proofreading of manuscript. All authors contributed to the article and approved the submitted version.
